# Sorption of Platinum and Palladium on Polyethylene Microplastics in Natural Water

**DOI:** 10.3390/molecules29245987

**Published:** 2024-12-19

**Authors:** Sylwia Sajkowska, Barbara Leśniewska

**Affiliations:** 1 Doctoral School, University of Bialystok, Ciołkowskiego 1K, 15-245 Białystok, Poland; s.sajkowska@uwb.edu.pl; 2 Department of Analytical and Inorganic Chemistry, Faculty of Chemistry, University of Bialystok, Ciołkowskiego 1K, 15-245 Bialystok, Poland

**Keywords:** Pt, Pd, aged PE-MP, sorption kinetics, sorption isotherms, lake water

## Abstract

In this work, for the first time, the sorption behaviour of platinum and palladium on polyethylene microplastics (PE-MP) was studied. To simulate natural conditions, part of PE-MP was subjected to the ageing process in lake water under the influence of solar radiation. The original and aged PE-MP was characterised using elemental analysis, FT-IR, SEM-EDX, and nitrogen porosimetry methods. The studies on Pt and Pd sorption on PE-MP were carried out in batch mode in natural lake water at pH 7.6. It was found that the ageing process led to the degradation of the surface of the PE-MP and the formation of a biofilm. The sorption process of Pt and Pd on PE-MP particles proceeds according to pseudo-second-order kinetics. A good fit of the experimental data to the Freundlich and Langmuir isotherm model indicates the mixed nature of Pt and Pd sorption on PE-MP. It was clearly indicated that Pt and Pd sorption from natural waters can occur on the surface of inert polyethylene particles, which can lead to the preconcentration of these elements, even from waters with a very low content, and transferring them over longer distances. This poses a threat to the health of living organisms and humans.

## 1. Introduction

The list of substances considered as environmental pollutants is constantly growing due to the analytical possibilities of their determination at very low levels, and studies proving their toxic effects on living organisms. In recent years, microplastics, i.e., plastic particles with a diameter of less than five millimetres, have been recognised as a new environmental pollutant. Their occurrence has been demonstrated in seawater, freshwater, sediments, soil, beach sand, and even polar zones [1]. Microplastics may adsorb pollutants such as heavy metals and pharmaceuticals on their surface. Therefore, the consumption of contaminated microplastic particles can pose a serious threat to animals and humans [1,2,3].

The widespread use of platinum group metals (PGM) over the past 40 years has led to the environmental pollution of these elements [4]. Due to their specific properties, they are used as industrial catalysts, in the manufacture of jewellery, as dental alloys (Pd), and in cancer therapy (Pt). However, the largest amounts of platinum group metals are used in the automotive industry to produce automotive catalysts [5]. Catalysts containing up to five grams per kilogram of platinum metals are used in motor vehicles to eliminate carbon oxides, hydrocarbons, and nitrogen oxides from exhaust fumes [6,7]. During vehicle operation, the catalytic converter is exposed to cracking and crumbling of the monolith due to high temperatures, rapid changes in oxidation-reduction conditions, erosion, and mechanical abrasion. The resulting catalyst particles, containing platinum group metals, enter the atmosphere with exhaust gases and are deposited in street dust, soil, and plants near roads and highways [6,7,8,9]. During precipitation, surface runoff from roads carries platinum group metals to natural water bodies, causing their accumulation in bottom sediments [7,8,10,11]. Elevated concentrations of platinum and palladium have been detected in various water samples such as rainwater, groundwater, surface water, seawater, marine sediments, sludge, etc. (see Table 1).

Significant sources of platinum in the environment are hospitals, where cancerostatic platinum compounds, predominantly cisplatin, carboplatin and oxaliplatin, are used in cancer therapy [12]. During treatment, the administered Pt-based drugs are eliminated with urine from the patient’s body and end up in sewage as highly active and cytotoxic forms. Unfortunately, it should be noted that Pt-based drugs are removed from hospital sewage through their adsorption in activated sludge of wastewater treatment plants only by 51–63% [12]. The lack of appropriate sewage treatment methods contributes to environmental pollution with platinum metal, especially in natural waters. Moreover, studies have shown that platinum-based drugs may have a toxic effect on aquatic organisms in urban sewers and receiving water bodies [12,13].

**Table 1:** Platinum and palladium content in selected freshwater reservoirs.

Reservoir	Content		Ref.
	Pt	Pd	
rainwater (Tokyo, Japan)	35–121 pg kg^−1^	-	[14]
rainwater (Seoul, South Korea)	3–66 pg kg^−1^	-	[15]
rainwater (Stuttgart, Germany)	-	<5.0 ng L^−1^	[11]
lake Rottenstone (Saskatchewan, Canada)	1.0–2.0 ng L^−1^	1.0–2.0 ng L^−1^	
river Rhine (Germany)	-	0.4 ng L^−1^	
estuary Volta river (Ghana)	12.0 ng L^−1^	17.0 ng L^−1^	[16]
river Göta älv (Gothenburg, Sweden)	-	10.2 ng L^−1^	
Hiko Spring (Nevada, USA)	0.2 ng L^−1^	<1.0 ng L^−1^	[11]
spring (Osaka, Japan)	-	22.0 ng L^−1^	
wastewater (Germany)	11–33 ng L^−1^	-	[16]
river sediment of Volta River estuary (Ghana)	7 µg kg^−1^	24 µg kg^−1^	
river sediment of Gironde estuary (France)	0.2–1.4 µg kg^−1^	-	[10]
lake sediment (Sheffield, United Kingdom)	3–14 µg kg^−1^	3–11 µg kg^−1^	[16]
sewage sludge (Sheffield, United Kingdom)	36 µg kg^−1^	80 µg kg^−1^	

In fresh waters (pH 6–8), platinum occurs mainly as Pt(II) in the form of neutral platinum(II) hydroxide Pt(OH)_2_, but depending on the conditions, Pt(IV) in the form of Pt(OH)_5_^−^ may also occur [17]. Palladium(II) occurs in the form of a neutral palladium(II) complex Pd(OH)_2_ [18]. Although there are similarities in properties, applications and thermodynamic equilibrium between platinum and palladium, the ligand exchange processes in the complexes of these metals proceed at different rates. For Pd(II), this process is very fast (within a few minutes), while for Pt(II) and Pt(IV) it takes longer (a few hours). Such differences in the kinetics of complexation processes lead to the so-called ageing of neutral Pt solutions [17]. In the platinum solution, where the metal occurs in the form of hexachloroplatinum(IV) anion [PtCl_6_]^2−^, with increasing pH, Pt(II) complexes are formed and coexist with Pt(IV) chloride complexes. With decreasing solution acidity during solution ageing, aquation and hydrolysis of the compounds occur, resulting in the formation of aquachloro-complexes and aquahydroxo-complexes of Pt(II) and Pt(IV) [17]. In estuarine waters, platinum and palladium may occur also as complex compounds with organic ligands. It is connected with a strong affinity of Pt and Pd ions to oxygenated carboxyl and phenolic groups occurring in humic acids (HA) present in water reservoirs (the stability constants of Pt and Pd complexes with humic acids are log *β* > 1) [18]. In the aquatic ecosystem, the presence of Pt and Pd, even in very low concentrations (Table 1), may influence aquatic organisms because these metals undergo bioaccumulation. The available literature has shown that platinum has much weaker bioavailability and bioaccumulation than palladium [13].

On the other hand, the presence of platinum metals in natural waters can lead to their sorption on the surface of microplastics, which are currently a common pollutant in the aquatic environment, both freshwater and marine water. Adsorption of metal ions on the surface of MP can occur due to electrostatic interactions or complexation reactions. This causes an increase in the concentration of metals on MP in relation to their trace amounts in waters, promoting their spread over longer distances and their transfer along food webs, bringing potential hazards to organisms and humans [19,20].

Mechanical degradation processes of MP, either under the action of UV or microorganisms, as well as the formation of new functional groups or biogenic coatings on the surface of MP, significantly affect the adsorption of metals. Microplastics that are subjected to the ageing process, as a result of degradation, reduce the size of their particles, which contributes to the increase of their adsorption capacity. It has also been shown that with increasing ageing time, the surface of microplastics becomes increasingly rougher and has more pores, which increases the adsorption of pollutants [3,21]. Another important factor influencing the adsorption of heavy metals on MP is dissolved organic matter (DOM). In the aquatic environment, organic matter usually occurs in the form of dissolved particles, and the characteristic substances of organic matter are humic acids (HA) and fulvic acids. Those compounds are usually negatively charged and cause the formation of a copolymer with MP [22,23]. Negatively charged HA molecules can also be adsorbed onto the surface of MP, leading to the increased electrostatic interaction between metal ions and the MP surface. On the other hand, complex formation between metal ions and FA may reduce their adsorption on MP.

Microplastics alone or by interacting with other pollutants (e.g., metals, pharmaceu-ticals) can adversely affect living organisms, causing, e.g., oxidative stress or genotoxic and neurotoxic effects [24,25]. Therefore, microplastics ingested by various aquatic organisms, such as zooplankton, mussels, oysters, corals, fish, turtles, and even seabirds, can induce a risk to their health. MP particles with contamination can not only cause mechanical damage but also introduce harmful substances by their desorption under acidic intestinal conditions into aquatic organisms and the food web. Moreover, if MP enters these organisms, it can pose a risk to human health. Microplastics ingested by aquatic animals can block sections of the digestive tract, block the intestines (through rupture of villi and fission of enterocytes), lead to the phagocytosis of organisms, and even death. Microorganisms bioaccumulate microplastics, which can affect their development and gene modification [1,25]. Nevertheless, depending on the conditions, tested organisms, and contaminants, the interaction of metals with MP may result in synergistic, antagonistic, or additive toxic effects [26]. Studies on the sorption of heavy metals such as Pb, Cd, Cu, and Cr on various microplastics can be found in the literature [27,28,29]. However, only one work has concerned the impact of Pd and microplastics on living organisms (mussels) [27].

Therefore, our work aimed to gain new knowledge on the possibility of the sorption of platinum and palladium on microplastics under conditions simulating their behaviour in natural surface waters. The original high-density polyethylene (HDPE) microplastic and MP subjected to the ageing process in Milli-Q water and lake water under the influence of solar radiation in the SolarBox chamber were used in the study. The sorption kinetics and sorption mechanisms of Pt and Pd were studied on the original and aged PE microplastic using different isotherm models. Due to the presence of platinum and palladium at very low concentrations in the environment, the electrothermal atomic absorption spectrometry (ETAAS) method was used to determine these analytes in solutions after sorption on microplastics. To our knowledge, this is the first study of the sorption of Pt and Pd on PE microplastic. This research may clarify the environmental risks of coexisting microplastics and platinum group metals in the aquatic environment.

## 2. Results and Discussion

### 2.1. Microplastic Characterisation

The obtained original and aged microplastics were examined using elemental analysis, FT-IR, SEM-EDX, and nitrogen porosimetry.

Elemental analysis enabled us to obtain information on the percentage amount of carbon, hydrogen, and oxygen in the original and aged PE-MP. The obtained results of this analysis are presented in Table 2. Each PE-MP contained carbon and hydrogen, and the aged microplastics additionally showed the presence of about 2% oxygen.

**Figure 1:**
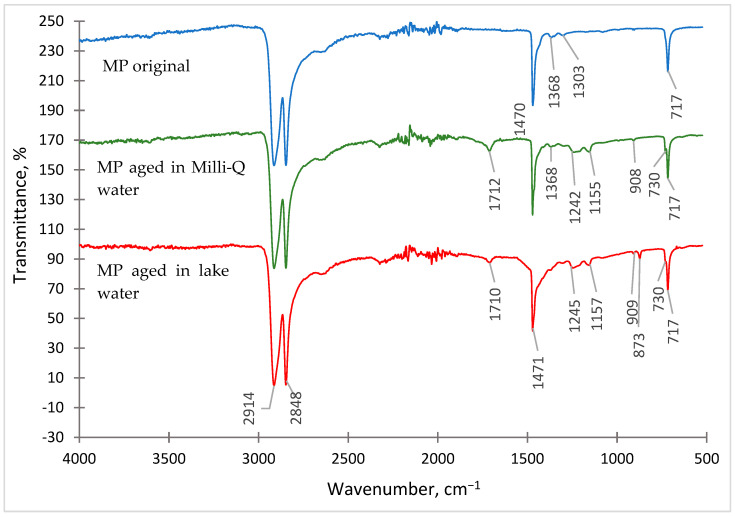
FT-IR spectrum of PE microplastic: original, aged in Milli-Q water, and aged in lake water.

In order to determine the presence of functional groups on the surface of PE microplastic, a Fourier-Transform Infrared Spectroscopy with a diamond reflectance attachment was used. The spectra of the original microplastic samples and those aged under different conditions were recorded (Figure 1).

The obtained FT-IR spectra of PE-MP are characterised by the presence of three intense bands of methylene groups (-CH_2_-), which appear as doublets at 730 cm^−1^ and 717 cm^−1^, 2914 cm^−1^ and 2848 cm^−1^ and as singlets at 1471 cm^−1^. The occurrence of other signals was also observed in the spectra of aged MP, which correspond to the vinyl group bands (-C=C-) at 908 cm^−1^ and 873 cm^−1^ and to the carbonyl group bands (-C=O) at 1712 cm^−1^ and 1710 cm^−1^. The presence of vinyl and carbonyl groups in high-density polyethylene may be related to the photooxidation process during irradiation in the ageing chamber [30,31].

FT-IR spectroscopy was also used to determine the extent to which PE-MP underwent degradation after ageing. For this purpose, its degree of crystallinity (*X*) was calculated using the following formula:

(1) $$X=100-\frac{1-\frac{I_{c}}{I_{a}}}{1.233\cdot \left(1+\frac{I_{c}}{I_{a}}\right)}\cdot 100$$

where:

- *I_c_*—signal intensity at 730 cm^−1^,
- *I_a_*—signal intensity at 720 cm^−1^,
- 1.233—theoretical value defining the *I_c_/I_a_* ratio at an angle of 45° [32].

The degree of crystallinity refers to the ratio of the mass of the crystalline part of the polymer to its total mass [1]. HDPE belongs to semicrystalline polymers and is characterised by crystallinity at the level of 70–95%, which depends on the density of the polymer (the denser the polymer, the higher the content of the crystalline part) [33,34]. Researchers [34] showed that HDPE not subjected to any ageing processes is characterised by crystallinity of 51.17%, which is consistent with the obtained results (Table 3). The increase in the degree of crystallinity in aged microplastics indicates that as a result of the photooxidation of HDPE, part of the amorphous region was degraded [35,36].

**Figure 2:**
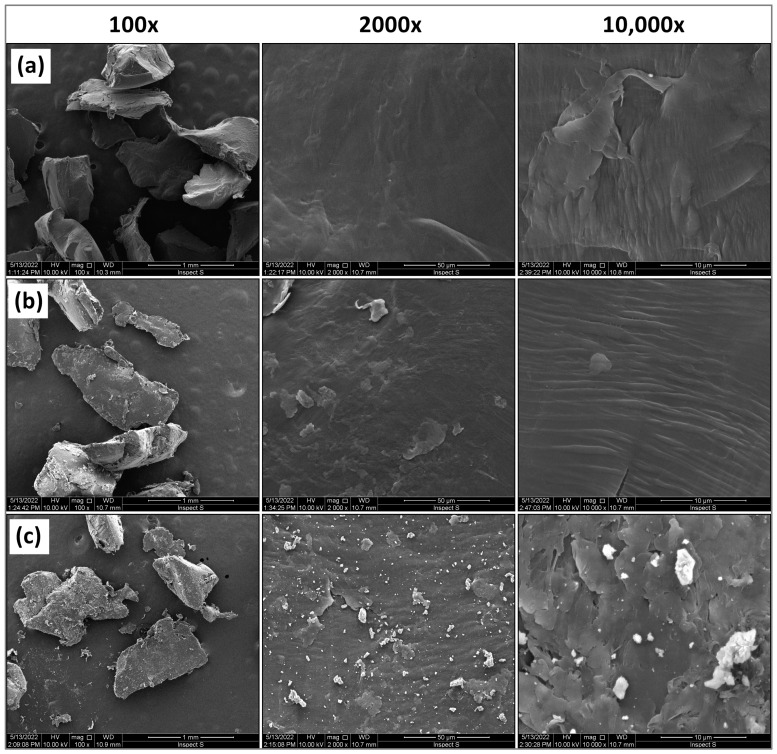
SEM images of MP surface: (**a**) original, (**b**) aged in Milli-Q water, (**c**) aged in lake water (particle size 500–1000 µm).

The SEM-EDX method was used to examine the surface morphology and elemental composition of the MP. Polyethylene is a poorly conductive material, and for this reason, it was sputtered with a 6 nm gold layer before measurement, and a potential of 10.0 kV was used for SEM measurements. Such preparation of the MP allowed for observation and taking pictures of its surface (Figure 2) using a scanning electron microscope at a magnification of up to 10,000 times.

**Figure 3:**
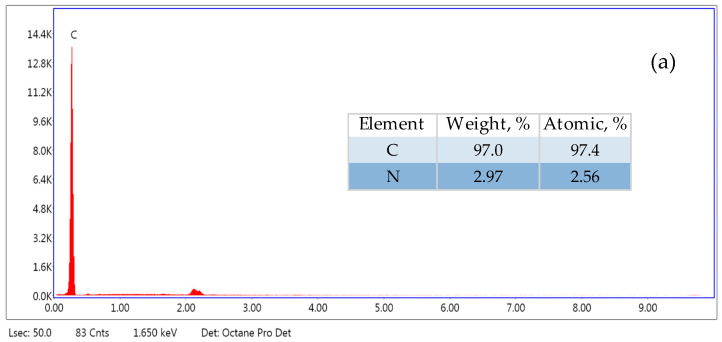

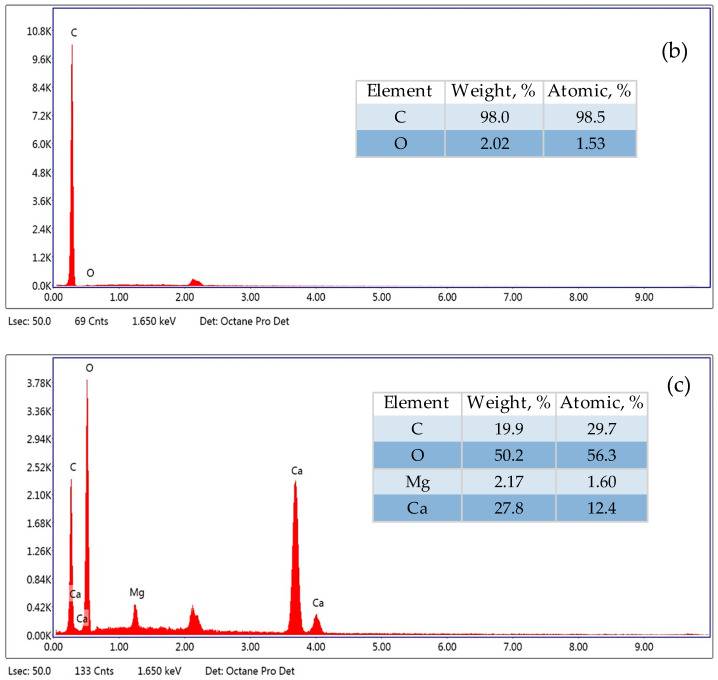
SEM–EDX spectra of PE microplastic (500–1000 µm): (**a**) original, (**b**) aged in Milli-Q water, (**c**) aged in lake water—white particle on its surface.

Based on the obtained images (Figure 2) at 100× magnification, it was found that the microplastic particles have irregular shapes. No cracks or pores were observed in the original microplastic and the one aged in Milli-Q water. A smooth surface characterises both microplastics. On the other hand, the MP aged in lake water underwent the degradation process to the greatest extent, as it has an uneven, cracked surface. Additionally, there are many light particles on its surface, the composition of which was examined by SEM–EDX (Figure 3).

SEM–EDX analysis allowed us to determine the elemental composition of microplastics. The studies showed that the original MP contains mainly carbon, while MP aged in Milli-Q water and in lake water contains oxygen in addition to carbon. In the case of microplastics aged in lake water, the composition of the substance present on its surface was also examined (Figure 3c). It contains carbon, oxygen, calcium and magnesium. The presence of these elements indicates that calcium and magnesium hydroxides could be crystallised on the surface of MP aged in lake water.

The porosity of microplastics was analysed using the nitrogen adsorption–desorption method at −196 °C. The specific surface area of the original and aged PE-MP was determined using Brunauer–Emmett–Teller (BET) analysis. The specific surface area of the original MP is 13.5 m^2^ g^−1^. The Milli-Q water-aged microplastic and lake water-aged PE-MP are characterised by a specific surface area of 69.9 m^2^ g^−1^ and 83.2 m^2^ g^−1^, respectively, which indicates that the ageing process can increase the specific surface area of the microplastic.

### 2.2. Characteristics of Lake Water

Water samples were collected four times in the period from March to May 2022 from the surface of Ełk Lake, which has a surface area of 3.82 km^2^ and is classified as the eighth largest lake in Poland in terms of depth. The average depth of this reservoir is 15 m, with the deepest area of 58.2 m. Lake water samples were filtered through a filter paper and tested for the parameters presented in Table 4.

Comparing the obtained results with the values given in the Regulation of the Polish Minister of Infrastructure [37], it was found that Ełk Lake can be described as a lake of class III purity, i.e., with a moderate ecological condition. The pH value in the range of 5.5–8.5 corresponds to the class II purity of surface waters, however, the obtained result of specific electrolytic conductivity is not consistent with class II (≤100 µS cm^−1^), which proves that this reservoir is a lake of the class III purity [37].

COD and BOD are the standard parameters for determining oxygen demand. In the case of COD, it is connected with oxygen consumption during the decomposition of organic matter and the oxidisation of inorganic matter. In the case of BOD, it is the concentration of oxygen demand required by microorganisms to decompose organic matter. Whereas, TOC is used to describe organic pollutants (carbon compounds) in water [38]. In the case of lake water, there are no specific standards for COD, BOD, and TOC. However, by comparing the obtained results for these parameters with the standards for purity of the surface water, we can say that water from Ełk Lake belongs to the II class of purity (BOD ≤ 6 mg L^−1^, COD ≤ 30 mg L^−1^, TOC ≤ 15 mg L^−1^) [37].

The lake water was used for the ageing of PE-MP under solar radiation and for the preparation of the Pt(IV) and Pd(II) solutions to study their sorption on PE-MP under conditions similar to those occurring in the environment.

### 2.3. Optimisation of the Sorption Conditions of Pt(IV) and Pd(II) on Microplastics

In order to investigate the possibility of sorption of platinum and palladium on PE microplastic, the sorption conditions as pH of the sample solution, the mass of microplastic, and contact time of the analyte with microplastic were optimised. The sorption of Pt and Pd from solutions on MP was calculated using the following formula:

(2) $$\%\;sorption=\frac{C_{0}-C_{e}}{C_{0}}\cdot 100\%$$

The masses of sorbed analytes at equilibrium (*q_e_*) and at time *t* (*q_t_*) were calculated using the following formula:

(3) $$q_{e},q_{t}=\frac{C_{0}-C_{e}}{m}\cdot V$$

where:

- *C*_0_ (ng mL^−1^)—initial concentration of the analyte in the solution without MP,
- *C_e_* (ng mL^−1^)—analyte concentration after sorption on MP,
- *m* (g)—a mass of microplastic,
- *V* (mL)—volume of platinum/palladium solution [39].

#### 2.3.1. The Influence of the pH of the Pt(IV) and Pd(II) Solution on the Sorption Efficiency of Analytes on Microplastics

**Figure 4:**
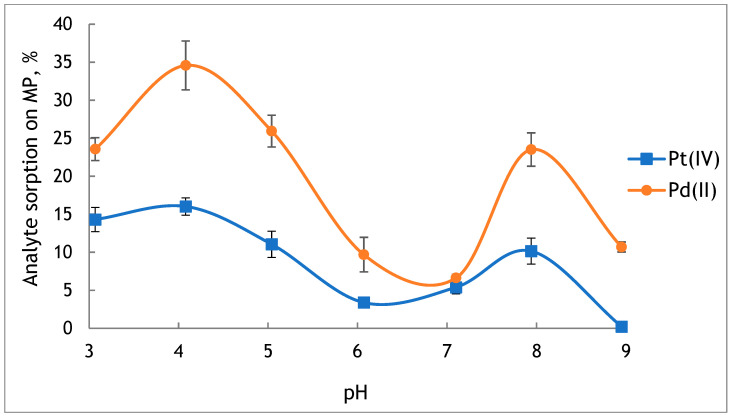
The effect of the pH value of the sample solution on the sorption of Pt(IV) and Pd(II) on the original PE-MP (value ± standard deviation, *n* = 3).

The pH of the sample has a major impact on the amount of analytes retained on microplastics, as platinum metals can occur in various forms in the aqueous environment. The tests were conducted to evaluate the extent to which platinum and palladium are retained on MP from solution at pH values from 3 to 9. Based on the concentration of analytes in the solutions after sorption, the efficiency of their sorption on MP was calculated according to the Equation (2). The obtained results are presented in Figure 4.

It was found that platinum and palladium sorbed poorly on the surface of MP. The highest values of sorption for Pd(II) (24–35%) and Pt(IV) (11–16%) were obtained from acidic solutions at pH 3–5 and in solution at pH 8 (24% for Pd(II), 10% for Pt(IV)). It may be caused by the lack of functional groups on the surface of virgin PE, which limits its sorption properties. However, the original PE-MP has a point of zero charge (PZC) at pH 6.6, indicating that in a solution below this value, the MP will be characterised by a positively charged surface [28]. It favours the occurrence of electrostatic interactions with anionic forms of Pt and Pd and increases their sorption on the MP. In a solution at pH < 7, the amount of Pt in the form of PtCl_6_^2−^ and PtCl_5_(H_2_O)^−^ increases with the decrease in pH [40]. Moreover, in solutions at pH < 4, the dominant form of Pd(II) is PdCl_3_^−^. Therefore, a higher sorption of Pt(IV) and Pd(II) was observed from the solutions at pH 3–5.

In freshwater, at pH 6–8, the MP from PE will have a negatively charged surface. Under such conditions, anions are expected to be repelled from negatively charged surfaces. However, at the interface, specific ionic effects can dominate over direct electrostatic interactions. As a result, negatively charged ionic species can be adsorbed on the negatively charged surface of the MP [40]. In a water environment, the degree of ionisation and distribution of Pt and Pd species depends on the pH of solution and the type of occurring substances. In fresh waters (pH 6–8), platinum occurs mainly as neutral platinum(II) hydroxide Pt(OH)_2_, but depending on the conditions, a form PtOH^+^, Pt(OH)_4_^2−^,Pt(OH)_5_^−^ may also occur [41]. Meanwhile, palladium occurs mostly as a neutral complex Pd(OH)_2_ [18]. In the presence of dissolved organic matter (DOM), Pt and Pd may form soluble complexes with humic and fulvic acids, which may affect their sorption on MP.

Based on the obtained results and the pH value of natural freshwaters, the pH of solutions in the range of 7–8 was chosen for further studies.

#### 2.3.2. The Influence of MP Mass on the Efficiency of Pt(IV) and Pd(II) Sorption on MP

**Figure 5:**
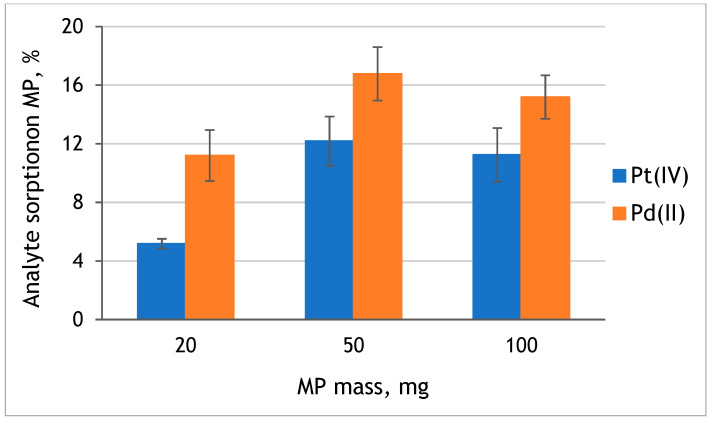
The effect of the mass of original PE-MP on the sorption of Pt(IV) and Pd(II) from solution at pH 8 (value ± standard deviation, *n* = 3).

The effect of the mass of microplastic on the efficiency of platinum and palladium sorption was examined with the use of 20, 50, and 100 mg of original MP and Pt(IV) and Pd(II) model solutions at pH 8. The obtained results are presented in Figure 5. It was observed that with the increase of the used mass of MP from 20 mg to 50 mg, Pd(II) and Pt(IV) sorption efficiency increased 1.5 and 2.3 times, respectively. However, the further increase of MP mass to 100 mg did not influence the sorption efficiency of analytes on MP. Therefore, 50 mg of PE-MP was used in further studies.

#### 2.3.3. Optimisation of Contact Time Pt(IV) and Pd(II) with Microplastics

The effect of Pt(IV) and Pd(II) contact time with MP on the sorption efficiency of these metals was investigated under conditions given in Table 5. The masses of sorbed analytes per unit mass of MP were calculated in the tested time range according to Equation (3) and the dependence of *q_t_* on the contact time of Pt(IV) and Pd(II) with MP was plotted in Figure S1.

Based on Figure S1, it was observed that the sorption process of Pt(IV) and Pd(II) on MP was initially very fast. In the case of both analytes, it was shown that after 15 min, significant amounts of Pt(IV) and Pd(II) were sorbed on all tested MP. With an extension of the contact time of the analytes with MP, the *q_t_* value increased more slowly until it reached a plateau, indicating that the state of dynamic equilibrium between the adsorbate and the adsorbent was achieved. The obtained times of reaching the equilibrium state and values of sorption capacity, *q_t_*, are presented in Table 5. For longer contact times of Pt(IV) and Pd(II) with MP, the sorption capacity did not increase because the availability of active sites decreased, which restricted further interaction with the analytes.

It can be seen that the Pt(IV) sorption on MP-a-MQ from the model solution occurs very quickly. However, after about 6 h, the desorption process occurs. In the case of solutions prepared in lake water, we can see a gradual increase in Pt(IV) sorption at the beginning and, with time, a plateau is formed, which indicates that the equilibrium state has been achieved. In the case of Pt(IV) sorption on the original MP and MP aged in Milli-Q water, the sorption equilibrium state was achieved after 48 h, and on MP aged in lake water, this state was achieved after 24 h. Faster achievement of the equilibrium state on MP aged in lake water may be due to the presence of a biofilm on its surface, on which Pt(IV) can also be sorbed. In the equilibrium state, more Pt(IV) from solutions prepared in lake water was sorbed on the original MP than on MP aged in lake water. The reason for the lower Pt(IV) sorption capacity on MP aged in lake water may be the fact that the surface of this MP is covered with calcium and magnesium hydroxides (Figure 2 and Figure 3), which could block the access of Pt(IV) to free active sites of MP.

In the case of Pd(II), the equilibrium of sorption was reached after 2 h on the original MP and MP aged in Milli-Q water. Meanwhile, on MP aged in lake water, this state was reached after 1 h, and a low sorption capacity of Pd(II) was obtained. It may be due to the presence of biofilm on the surface of the aged MP, which limits access to its active sites. At equilibrium, more Pd(II) from solutions prepared in lake water was sorbed onto the original MP than onto MP aged in lake water. This indicates a significant effect of the microplastic surface on Pd(II) sorption. The low value of Pd(II) sorption capacity on MP-a-LW results from covering the microplastic surface with calcium and magnesium hydroxides (Figure 2 and Figure 3), which limits the number of active sites of MP-a-LW. In addition, negatively charged organic matter could limit the sorption of Pd(II) onto the biofilm formed on MP aged in lake water. Moreover, Zhou et al. [39] showed that with the increase of fulvic acid concentration, the adsorption of metals on MP is clearly stopped, which may indicate the possibility of forming complexes between metal ions and fulvic acid. The formation of Pd(II) complexes with DOM in lake water was less probable because the high value of Pd(II) sorption capacity from such a solution was obtained on the original MP.

In the case of MP aged in Milli-Q water, it can be observed that when the same concentrations of analytes were used in model solutions, more palladium than platinum was sorbed at equilibrium. The dynamic equilibrium was also achieved faster for Pd(II) than for Pt(IV). The obtained results may be due to the smaller ionic radius of Pd (Pd^2+^ 0.64 Å and Pd^4+^ 0.63 Å) than Pt (Pt^2+^ 0.80 Å and Pt^4+^ 0.62 Å) [42] and the possibility of the occurrence of different forms of these analytes in the solution.

### 2.4. Sorption Kinetics

**Figure 6:**
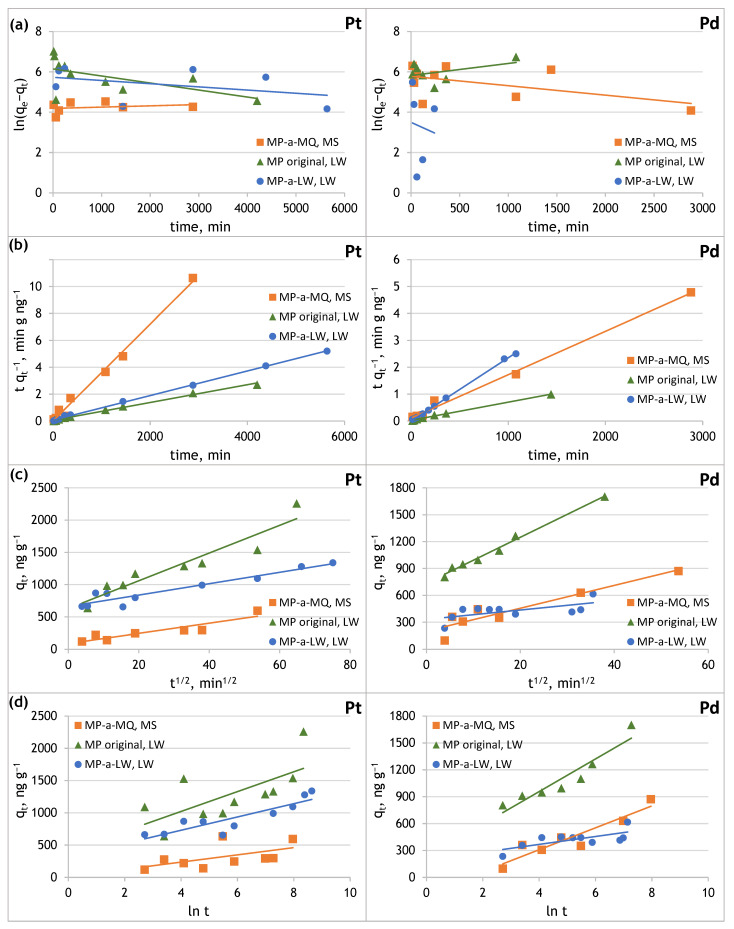
Sorption kinetics of Pt(IV) and Pd(II) onto original and aged microplastics in Milli-Q and lake water: (**a**) pseudo-first-order model, (**b**) pseudo-second-order model, (**c**) intra-particle diffusion model, (**d**) Elovich model.

To determine the mechanism of Pt(IV) and Pd(II) sorption on PE microplastic, the experimental data on the time dependence of analyte sorption were substituted into pseudo-first-order, pseudo-second-order, intraparticle diffusion, and Elovich reaction models and the appropriate kinetic parameters were determined (Table 6). The obtained fitting plots of the sorption kinetics of Pt(IV) and Pd(II) onto original and aged PE microplastic are presented in Figure 6.

The sorption of Pt(IV) and Pd(II) on original and aged PE microplastic can be adequately described using the pseudo-second-order kinetic model, rather than by the pseudo-first-order kinetic model. This is proved by the obtained linear coefficient of determination *R*^2^, which was close to one, rectilinear dependencies, and good agreements of *q_t_* with *q_e_* in the pseudo-second-order kinetic model. Chemisorption may occur during Pt(IV) and Pd(II) sorption on PE microplastic. The determined pseudo-second-order rate constants (*k*_2_) show that Pt(IV) sorption is fast on MP aged in Milli-Q water and Pd(II) on MP aged in lake water, while Pt(IV) and Pd(II) sorption is slow on the original MP.

The fitting for the intraparticle diffusion and Elovich kinetic model was not as close as that achieved with the pseudo-second-order kinetic model. In the case of the intraparticle diffusion model, the coefficient of determination ranges from 0.8380 to 0.8978 for Pt(IV), and from 0.4169 to 0.9897 for Pd(II). The *C_i_* values were higher than zero for all cases studied, which indicates that none of the straight lines passed through the origin. It may indicate that the rate-limiting step of the adsorption process is not dominated by intra-particle diffusion, but is instead controlled by surface adsorption, diffusion within the pores, and diffusion in liquid films [29,43]. The coefficient of determination obtained from the Elovich kinetic model ranges from 0.3150 to 0.7762 for Pt(IV), and from 0.5077 to 0.8865 for Pd(II). The Elovich kinetic model shows higher initial sorption rate (*α*) values for PE-MP immersed in platinum and palladium solutions prepared from lake water, rather than immersed in model solutions of these metals.

### 2.5. Sorption Isotherms

**Figure 7:**
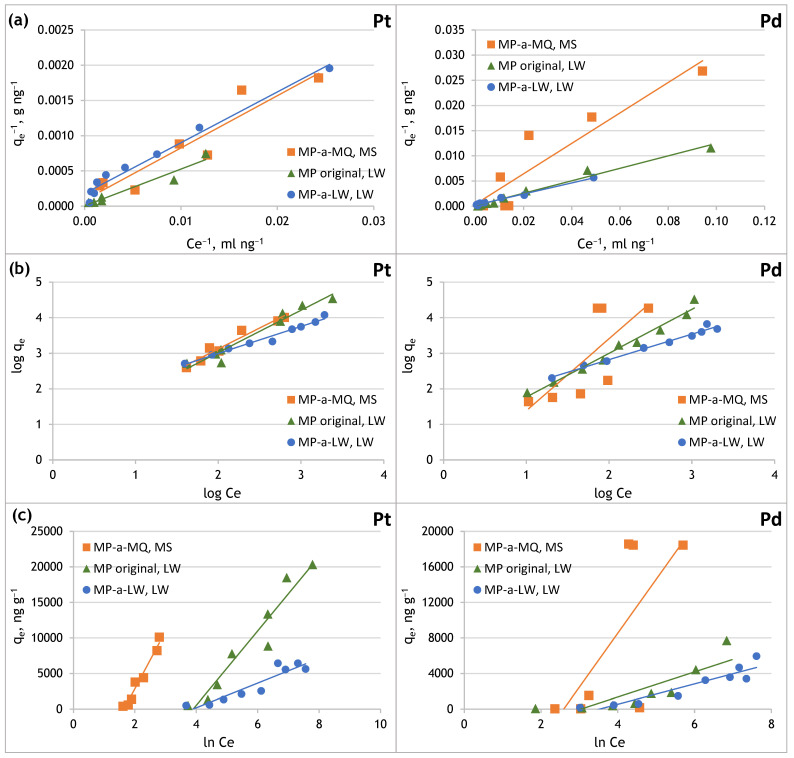
Sorption isotherms of Pt(IV) and Pd(II) onto original and aged microplastics in Milli-Q and lake water: (**a**) Langmuir model, (**b**) Freundlich model, (**c**) Temkin model.

To characterise the sorption processes of Pt(IV) and Pd(II) on MP, studies were carried out to determine the sorption isotherm model of these analytes and the values of the parameters characterising each of the Langmuir, Freundlich, and Temkin isotherms models. The experimental data and the corresponding determination coefficients (*R*^2^) for each model can be found in Table 7. Figure 7 shows the determined Langmuir, Freundlich, and Temkin isotherm models of Pt(IV) and Pd(II) sorption on original and aged MP.

The determined Langmiur isotherm parameter *R_L_* (partition coefficient, equilibrium parameter) indicates the affinity between the adsorbate and the adsorbent. The value of the *R_L_* parameter determines whether the adsorption process is unfavourable (*R_L_* > 1), linear (*R_L_* = 1), favourable (0 < *R_L_* < 1) or irreversible (*R_L_* = 0). The *R_L_* parameter is calculated according to the following equation:

(4) $$R_{L}=\frac{1}{1+K_{L}C_{0}}$$

where:

- *K_L_* (mL ng^−1^)—Langmuir isotherm constant,
- *C*_0_ (ng mL^−1^)—initial concentration of adsorbate in solution.

In the case of sorption of Pt(IV) and Pd(II) on the original PE microplastic and the MP aged in Milli-Q water and in lake water, the obtained partition coefficient is in the range of 0–1, which indicates that the process of both metals’ sorption on PE-MP is favourable.

Based on the plots made (Figure 7) a linear relationship was obtained in the case of all isotherm models, indicating that the Pt(IV) sorption process on the original MP and the aged in Milli-Q water and lake water MP can be described using these adsorption models. The linear correlation coefficients close to unity (Table 7) indicated that the Langmuir, Freundlich, and Temkin isotherm models fit the experimental data well, which may indicate a mixed nature of the Pt(IV) sorption process on microplastics. However, it can be assumed that on the original PE microplastic, due to the homogeneous surface of this material, Pt(IV) sorption occurs as a monolayer, which is consistent with the Langmuir model. On the other hand, in lake water, Pt(IV) complexation can occur in the presence of organic matter, and the resulting forms can interact with the adsorbed layer of analytes, which indicates multilayer adsorption consistent with the Freundlich model. In the case of PE microplastic aged in lake water, its surface is inhomogeneous due to degradation and biofilm formation. Therefore, the Freundlich model better describes Pt(IV) sorption from lake water, as evidenced by the high value of the Freundlich constant. In the case of PE microplastic aged in Milli-Q (which has a homogeneous surface), the linear correlation coefficient is higher for the Freundlich and Temkin isotherms than for the Langmuir isotherm.

In the case of Pd(II), it was found that the sorption of this metal on the original MP and MP aged in lake water is well described by the Langmuir and Freundlich models. The sorption of Pd(II) on MP does not follow the Temkin model, as indicated by the low values of the linear coefficient of determination. In contrast to platinum, palladium sorption on MP aged in Milli-Q water is better described by the Langmuir model. However, further research is needed to explain the differences in the isotherm patterns of platinum and palladium.

The *q_max_* values were determined for the original and aged PE microplastic. The lower Pt(IV) and Pd(II) sorption capacity on the MP aged in lake water than in the original MP may result from covering its surface with calcium and magnesium compounds, which could block Pt(IV) and Pd(II) access to a more significant number of active sites of the MP. Additionally, negatively charged organic matter could have been retained on the MP’s surface, which limited Pt(IV) and Pd(II) sorption on the biofilm formed on the MP aged in lake water. Pt(IV) and Pd(II) sorption from lake water to the original MP may occur as a monolayer. In the case of MP aged in lake water, Pt(IV) and Pd(II) sorption on its heterogeneous surface may occur in multiple layers. The high values of the Freundlich constant confirm this.

## 3. Materials and Methods

### 3.1. Reagents

Stock solutions of Pt(IV) and Pd(II) (1000 μg mL^−1^ in 10% HCl, Inorganic Ventures, Christiansburg, VA, USA) were used for the preparation of working standard solutions. Hydrochloric acid (35%, TraceSelect, Fluka, Paris, France) and sodium hydroxide (Chempur, Piekary Śląskie, Poland) were used to adjust the pH of samples and standards. Solutions were prepared using ultrapure water obtained from a Milli-Q system (Direct-Q3; Merck Millipore, Darmstadt, Germany). The reference material of synthetic water CRM water 3 (CPAchem, Bogomilovo, Bulgaria) was used for quality control of measurements.

Lake water samples were collected from the Ełk Lake (Ełk, Poland). All samples were filtered on filter paper and stored at 2 °C. According to standard methods, some properties of collected water samples, such as pH, conductivity, Total Organic Carbon (TOC), Chemical Oxygen Demand (COD), and Biochemical Oxygen Demand (BOD), were determined. The ICP multi-element standard solution VIII (100 mg L^−1^ of 24 elements in 2% HNO_3_, Certipur, Merck, Darmstadt, Germany) was used for the preparation of working standard solutions of selected metals prior to the analysis of lake water samples using the inductively coupled plasma mass spectrometry method (ICP-MS). Rhodium standard for ICP-MS (1000 μg mL^−1^ in 2% HNO_3_, Sigma-Aldrich, St. Louis, MO, USA) was used to prepare an internal standard solution at a concentration of 100 ng mL^−1^. Nitric acid (>69.5%, Trace Select, Fluka, Dresden, Germany) was used for the preparation of the sample and standards.

### 3.2. Preparation of Microplastic

Virgin polyethylene microplastics were prepared from high-density polyethylene (HDPE) pellets (Sigma-Aldrich, St. Louis, MO, USA) by grinding them in a cryogenic mill (6875 Freezer/Mill, SPEX SamplePrep, Metuchen, NJ, USA) at liquid nitrogen temperature (−196 °C). The obtained microplastic was then separated into individual fractions using the Vibratory Sieve Shaker (Analysette 3, Fritsch, Idar-Oberstein, Germany), and in further studies, a fraction with a particle size of 500–1000 µm was used. Part of the PE microplastic was subjected to the ageing process in the ageing chamber (SolarBox 3000e, CO.FO.ME.GRA., Milano, Italy) for 17.5 days (420 h) at a radiation intensity of 1000 W m^−2^ and a temperature of 35 ± 2 °C. The ageing chamber was equipped with a xenon lamp and a standard sun filter with a wavelength of 300–800 nm. The microplastics have been aged under two different conditions: (1) in Milli-Q water (MP-a-MQ) and (2) in lake water from Ełk Lake (MP-a-LW).

### 3.3. Instrumentation

For the determination of platinum and palladium concentration in gained solutions, an atomic absorption spectrometer with electrothermal atomisation (Solaar M 650983 v1.30, Thermo Electron Corporation, Altrincham, UK) was used. The measurements were done at wavelengths λ = 266.1 nm (Pt) and 247.7 nm (Pd) with a slit of 0.5 nm and sample volume 20 µL. The optimized furnace heating programme is shown in Table 8.

A Triple Quadrupole ICP-MS (8800 ICP-QQQ, Agilent Technologies, Singapore) fitted with a MicroMist nebulizer, Scott-type double pass spray chamber Peltier cooled, nickel sampler and skimmer cones, and collision/reaction cell (octopole reaction system ORS^3^) was used for the determination of Ca, Na, Mg, K, Zn, Cu, Fe, Al, Mn, and Cr in lake water samples. For the elimination of the polyatomic interferences, helium was used as a collision gas.

The conductivity of the water samples from Ełk Lake was tested with a conductometer CPC-505 (Elmetron, Zabrze, Poland). The pH values of solutions were controlled with a pH meter (inoLab pH Level 1, WTW, Weilheim, Germany) equipped with a pH electrode (SenTix 21, WTW, Weilheim, Germany). A shaking water bath (SWB 22N, LaboPlay, Bytom, Poland) was used for the study of Pt and Pd sorption on PE microplastic.

An Elemental Analyser was used for the analysis of C, H, and O content in PE microplastic (Vario Micro Cube, Elementar, Langenselbold, Germany). The FTIR spectra of microplastics were acquired using an Attenuated Total Reflectance Fourier Transform Infrared (ATR-FTIR) spectrometer (Nicolet 6700, Thermo Scientific, Waltham, MA, USA). The spectra were performed in the spectral range from 500 to 4000 cm^−1^ at room temperature.

The surface morphology of the original and aged microplastics was observed by scanning electron microscopy (SEM, Inspect S50, FEI, Hillsboro, OR, USA). Energy dispersive spectroscopy (EDS, FEI, Lexington, KY, USA) was used to analyse the distribution of the elements on the MP. Before measurement, the microplastics were sputtered with 6 nm of a gold layer using the Low Vacuum Coater (EM ACE200, Leica Microsystems, Wetzlar, Germany). Images were recorded at 10 kV acceleration voltages and with a magnification of 100, 2000, and 10,000 times.

The specific surface area of original and aged PE microplastic in Milli-Q water and lake water was determined using the nitrogen adsorption–desorption technique at −196 °C using an adsorption analyser (Gemini VII 239, Micromeritics, Norcross, GA, USA). Each sample was degassed before analysis. Specific surface areas were calculated using the BET equation.

### 3.4. Sorption of Platinum and Palladium on Microplastics

#### 3.4.1. Optimisation of Sorption Conditions of Pt(IV) and Pd(II) on MP

During the optimisation of the sorption conditions of Pt(IV) and Pd(II) on MP, the pH of the sample solution and the microplastic mass were examined. To study the influence of the pH of the analytes solutions on their sorption on MP, 100 mg of the original MP were weighed into glass test tubes, and 5 mL of a solution containing 100 ng mL^−1^ Pt(IV) or 50 ng mL^−1^ Pd(II) in the pH range from 3 to 9 was added. To obtain the desired pH value, NaOH and HCl solutions of appropriate concentrations and volumes were added to the initial solution. The samples were then shaken for 1 h at a shaking speed of 150 rpm at 20 °C. After this time, the samples were passed through a separator to separate the microplastics from the solutions. The absorbance of Pt and Pd in the obtained solutions was measured using the ETAAS method.

In the next step, the effect of microplastic mass on the efficiency of platinum and palladium sorption was examined. For this purpose, 20, 50, and 100 mg of original MP were weighed into glass test tubes, and then 5 mL of a solution containing 50 ng mL^−1^ of Pt(IV) or 50 ng mL^−1^ of Pd(II) at pH 8 was added. The samples were then shaken for 2 h at a shaking speed of 150 rpm at 20 °C. The concentrations of Pt and Pd in the solutions after the separation of MP were determined using the ETAAS method. The sorption of analytes on MP was calculated based on calibration graphs.

#### 3.4.2. Sorption Kinetics and Sorption Isotherms Experiments

Under optimal conditions, sorption kinetics studies of platinum and palladium on PE-MP were carried out, and sorption isotherms were determined. The contact time required to achieve the state of dynamic sorption equilibrium between analytes and MP was specified. The pseudo-first-order, pseudo-second-order, intraparticle diffusion, and Elovich kinetic equations and Langmuir, Freundlich, and Temkin sorption isotherm models were determined.

During the studies, model solutions with appropriate concentrations of Pt(IV) and Pd(II) in Milli-Q water were used, which were prepared from Pt(IV) and Pd(II) stock solutions (1000 μg mL^−1^ in 10% HCl) using successive dilutions and adjusted to pH 8.0 with NaOH. Pt(IV) and Pd(II) solutions in lake water from Ełk Lake, prepared using successive dilutions of analyte stock solutions with a concentration of 1000 μg mL^−1^, were also used to simulate environmental conditions. In this case, the natural pH of the lake water was maintained at 7.6. The studies were performed in static mode using 5 mL of a platinum(IV) or palladium(II) solution and 50 mg of PE-MP.

The first experiment was conducted to determine the impact of the contact time of analytes with MP on the sorption efficiency and to evaluate the time required to achieve the dynamic equilibrium between the analytes and MP. For that purpose, 50 mg of original MP, MP aged in Milli-Q water, and MP aged in lake water were weighed into 25 mL glass test tubes. Then, 5 mL of 50 ng mL^−1^ Pt(IV) solution in Milli-Q water (pH 8) was added to the test tubes with aged MP in Milli-Q water. In the case of the original MP and the MP aged in lake water, 5 mL of 100 ng mL^−1^ Pt(IV) in lake water (pH 7.6) was added. Analogous samples of MP were prepared with 50 ng mL^−1^ of Pd(II) in Milli-Q water and lake water. The prepared samples were then shaken in a water bath at room temperature at a shaking speed of 150 rpm for 15, 30, 60, 120, 240, 360, 1080, 1440, and 2880 min in the case of the 50 ng mL^−1^ Pt(IV) and Pd(II) solutions, and 15, 30, 60, 120, 240, 360, 1440, and 2880, 4380, 5640 min in the case of the 100 ng mL^−1^ Pt(IV) solutions. Each measurement series contained four parallel samples and three comparative solutions (analyte solutions of the same concentration and pH without MP shaken for the same time). Then, MP was separated from solutions after sorption in a separator, and the concentrations of analytes in the obtained solutions were determined using the ETAAS method. When calculating the sorption efficiency, the initial concentrations of the Pt(IV) and Pd(II) were assumed to be the concentrations in the comparative solutions, which allowed for taking into account the losses of the analytes associated with their sorption on the vessel walls during long-term shaking.

The adsorption isotherms experiments were carried out with a series of concentrations of Pt(IV) (50, 100, 250, 500, 1000, and 2000 ng mL^−1^) and Pd(II) (10, 20, 30, 50, 100, 150, 250, 500, 800, 1000, 1200, 1500, and 2000 ng mL^−1^) in model solutions (pH = 8) and lake water (pH = 7.6). MP (50 mg) were weighed into 25 mL glass test tubes and 5 mL of a solution with a variable concentration of Pt(IV) (50–2000 ng mL^−1^) or Pd(II) (10–2000 ng mL^−1^) was added. Then, the samples were shaken in a water bath at room temperature at a shaking speed of 150 rpm for 2 h (Pd), 24 h (Pt, MP-a-LW) and 48 h (Pt, MP original, MP-a-MQ). After the separation of MP, the concentrations of analytes in the obtained solutions were determined using the ETAAS method.

### 3.5. Data Analysis

The experimental data, sorption kinetics and isotherm model formula fitting and data charts were analysed using the software Excel 2016 (Copyright Microsoft Excel 2016, Redmond, WA, USA). All data were presented as the mean value and standard deviation (SD, *n* = 4). The linear model fitting formulas are as follows:

(1) Pseudo-first-order kinetics formula:

(5) $$ln\left(q_{e}-q_{t}\right)=ln\;q_{e}-k_{1}t$$

(2) Pseudo-second-order kinetics formula:

(6) $$\frac{t}{q_{t}}=\frac{1}{k_{2}q_{e}^{2}}+\frac{1}{q_{e}}t$$

(3) Intraparticle diffusion model formula:

(7) $$q_{t}=k_{id}t^{1/2}+C_{i}$$

(4) Elovich kinetics model formula:

(8) $$q_{t}=\frac{1}{\beta }\ln\left(\alpha \beta \right)+\frac{1}{\beta }ln\;t$$

where:

- *q_e_* (ng g^−1^)—amount of adsorbate sorbed at equilibrium,
- *q_t_* (ng g^−1^)—amount of adsorbate sorbed at instant time *t* (min),
- *k*_1_ (min^−1^)—pseudo-first-order kinetics rate constant,
- *k*_2_ (g ng^−1^ min^−1^)—pseudo-second-order kinetics rate constant,
- *k_id_* (ng g^−1^ min^−1/2^)—intraparticle diffusion rate constant,
- *C_i_* (ng g^−1^)—parameter connected with boundary layer thickness of microplastic,
- *α* (ng g^−1^ min^−1^)—initial sorption rate in the Elovich model,
- *β* (g ng^−1^)—constant related to the extent of surface coverage and activation energy for chemisorption in the Elovich model.

(5) Langmuir model formula:

(9) $$\frac{1}{q_{e}}=\frac{1}{K_{L}q_{max}}\cdot \frac{1}{C_{e}}+\frac{1}{q_{max}}$$

(6) Freundlich model formula:

(10) $$log\;q_{e}=log\;K_{f}+\frac{1}{n}log{\;C}_{e}$$

(7) Temkin model formula:

(11) $$q_{e}={B\;ln\;K}_{T}+B\;ln{\;C}_{e}$$

where:

- *K_L_* (mL ng^−1^)—Langmuir isotherm constant,
- *q_max_* (ng g^−1^)—the maximum amount of adsorbate sorbed,
- *C_e_* (ng mL^−1^)—equilibrium concentration of adsorbate,
- *K_f_* ((ng g^−1^)(mL ng^−1^)^1/*n*^)—Freundlich isotherm constant,
- *n*—the empirical parameter relating to the adsorption intensity, which varies with the heterogeneity of the material (dimensionless),
- *B* (J mol^−1^)—constant related to the heat of sorption,
- *K_T_* (mL ng^−1^)—Temkin isotherm equilibrium binding constant.

## 4. Conclusions

In this study, the sorption behaviour of platinum and palladium on original, aged in Milli-Q water, and aged in lake water polyethylene microplastics was studied. At the pH of natural water, the sorption of Pt(IV) and Pd(II) on the original PE-MP occurred with low efficiency due to the forms of occurrence of analytes and the surface charge of PE-MP, but water acidification can enhance that process. The ageing process in natural lake water under the influence of solar radiation led to the degradation of the microplastic surface and the formation of a biofilm. The conducted studies showed that the sorption process of platinum and palladium on microplastic particles proceeds according to pseudo-second-order kinetics, which proves that the chemisorption of analytes on PE-MP dominates. A good fit of the experimental data to the Freundlich and Langmuir isotherm model indicates the mixed nature of Pt(IV) and Pd(II) sorption on PE-MP. The high value of the Freundlich constant confirms that multilayer adsorption of analytes occurs on the heterogeneous surface of MP aged in lake water. In the case of PE-MP aged in Milli-Q water, the monolayer sorption of analytes was better described by the Langmuir isotherm model. The comparison of the sorption capacity of analytes obtained in lake water on original and aged microplastics in lake water shows a much greater effect of the microplastic surface than the forms of Pt(IV) and Pd(II) occurrence on the amount of sorbed pollutants. The studies conducted clearly indicate that Pt(IV) and Pd(II) sorption from natural waters can occur on the surface of inert PE microplastic, which poses a risk of preconcentrating these elements even from waters with very low content and transferring them over longer distances.

Further studies of Pt and Pd sorption on microplastics, taking into account the variability of environmental conditions, are necessary to better predict the behaviour of those metals in a natural environment.

## Figures and Tables

**Table 2:** Elemental analysis results of original and aged PE microplastic under different conditions.

Type of MP	Content ± SD, %, *n* = 3		
	C	H	O
original	84.55 ± 0.05	15.45 ± 0.02	0
aged in Milli-Q water	82.70 ± 1.35	15.07 ± 0.31	2.23 ± 0.26
aged in lake water	82.65 ± 0.15	15.03 ± 0.05	2.32 ± 0.20

**Table 3:** Calculated degrees of crystallinity of PE microplastic.

Type of MP	Degree of Crystallinity, %
original	56.8
aged in Milli-Q water	64.2
aged in lake water	57.5

**Table 4:** Characteristics of lake water used for microplastic ageing and preparation of Pt(IV) and Pd(II) sample.

Parameter	Value
pH	7.63 ± 0.24
Conductivity	345–443 µS cm^−1^
TOC *	7.91 mg L^−1^
COD	16.4 mg L^−1^
BOD	5 mg L^−1^
Ca	35.1 µg mL^−1^
Na	17.1 µg mL^−1^
Mg	16.2 µg mL^−1^
K	6.39 µg mL^−1^
Zn	1.52 µg mL^−1^
Cu	0.95 µg mL^−1^
Fe	0.48 µg mL^−1^
Al	0.16 µg mL^−1^
Mn	0.028 µg mL^−1^
Cr	0.015 µg mL^−1^

* TOC—Total organic carbon, COD—chemical oxygen demand, BOD—biochemical oxygen demand.

**Table 5:** The conditions used to study the effect of Pt(IV) and Pd(II)’s contact time with MP on their sorption efficiency, the obtained times of reaching the equilibrium state of Pt(IV) and Pd(II) sorption on MP, and the sorption capacity of Pt(IV) and Pd(II) at time t.

MP and Solution Type	Analyte Concentration, ng mL^−1^	Contact Time, min	Time to Reach Equilibrium State, h	q_t_, ng g^−1^
MP-a-MQ, MS at pH 8.0	50 (Pt)	15–2880	48	299
	50 (Pd)		2	616
MP original, LW at pH 7.6	100 (Pt)	15–4200	48	1540
	50 (Pd)	15–1440	2	1307
MP-a-LW, LW at pH 7.6	100 (Pt)	15–5640	24	1079
	50 (Pd)	15–1260	1	444

MP-a-MQ—microplastic aged in Milli-Q water, MS—model solution (Milli-Q water), LW—lake water, MP-a-LW—microplastic aged in lake water.

**Table 6:** Sorption kinetics of Pt(IV) and Pd(II) onto original and aged microplastics in Milli-Q and lake water.

Analyte	Kinetic Model	Parameters	MP and Solution Type		
			MP-a-MQ, MS	MP Original, LW	MP-a-LW, LW
Pt	Pseudo-first order	*k*_1_, min^−1^ *q_e_*, ng g^−1^ *R*^2^	6.0 ∙ 10^−5^ 66 0.0594	3.0 ∙ 10^−4^ 467 0.3482	2.0 ∙ 10^−4^ 308 0.1705
	Pseudo-second order	*k*_2_, g ng^−1^ min^−1^ *q_e_*, ng g^−1^ *q_t_*, ng g^−1^ *R*^2^	1.3 ∙ 10^−3^ 278 299 0.9914	8.3 ∙ 10^−6^ 1429 1540 0.9936	1.2 ∙ 10^−5^ 1111 1079 0.9989
	Intraparticle diffusion	*k_id_*, ng g^−1^ min^−1/2^ *C_i_*, ng g^−1^ *R*^2^	7.87 88.2 0.8380	21.5 631 0.8922	8.84 662 0.8978
	Elovich	*α*, ng g^−1^ min^−1^ *β*, g ng^−1^ *R*^2^	68.3 0.0177 0.3150	2184 0.0065 0.4662	2266 0.0097 0.7762
Pd	Pseudo-first order	*k*_1_, min^−1^ *q_e_*, ng g^−1^ *R*^2^	5.0 ∙ 10^−4^ 325 0.2913	6 ∙ 10^−4^ 338 0.1993	2.2 ∙ 10^−3^ 33 0.0101
	Pseudo-second order	*k*_2_, g ng^−1^ min^−1^ *q_e_*, ng g^−1^ *q_t_*, ng g^−1^ *R*^2^	2.2 ∙ 10^−5^ 625 616 0.9944	1.7 ∙ 10^−5^ 1429 1307 0.9937	3.8 ∙ 10^−3^ 417 444 0.9991
	Intraparticle diffusion	*k_id_*, ng g^−1^ min^−1/2^ *C_i_*, ng g^−1^ *R*^2^	12.7 202 0.8790	25.6 736 0.9897	5.22 332 0.4169
	Elovich	*α*, ng g^−1^ min^−1^ *β*, g ng^−1^ *R*^2^	27.9 0.0082 0.8722	641 0.0055 0.8865	3439 0.0227 0.5077

**Table 7:** Fitting parameters for the Langmuir, Freundlich, and Temkin models.

Analyte	Isotherm	Parameters	MP And Solution Type		
			MP-a-MQ, MS	MP Original, LW	MP-a-LW, LW
Pt	Langmuir	*q_max_*, ng g^−1^ *K_L_*, mL ng^−1^ *R*_L_ *R*^2^	11,111 1.2 ∙ 10^−3^ 0.790 0.8811	200,000 9.6 ∙ 10^−5^ 0.948 0.9399	5000 2.8 ∙ 10^−3^ 0.415 0.9783
	Freundlich	*n* *K_f_*, (ng g^−1^)(mL ng^−1^)^1/*n*^ *R*^2^	0.8532 5.99 0.9549	0.841 4.40 0.9510	1.32 30.3 0.9748
	Temkin	*B*, J mol^−1^ *K_T_*, mL ng^−1^ *R*^2^	8099 0.193 0.9620	5231 0.020 0.9319	1731 0.021 0.8668
Pd	Langmuir	*q_max_*, ng g^−1^ *K_L_*, mL ng^−1^ *R_L_* *R*^2^	2500 1.3 ∙ 10^−3^ 0.842 0.8456	10,000 8.1 ∙ 10^−4^ 0.817 0.9812	5000 1.8 ∙ 10^−3^ 0.574 0.9910
	Freundlich	*n* *K_f_*, (ng g^−1^)(mL ng^−1^)^1/n^ *R*^2^	0.494 0.23 0.5488	0.812 3.46 0.9789	1.41 25.3 0.9817
	Temkin	*B*, J mol^−1^ *K_T_*, mL ng^−1^ *R*^2^	6057 0.075 0.4998	1411 0.048 0.7250	1142 0.030 0.8635

**Table 8:** Graphite furnace heating programme for the determination of platinum and palladium in solutions using ETAAS.

Stage of the Programme	Temperature, °C	Hold Time, s	Ramp Time, °C s^−1^
Drying	120	20	10
Pyrolysis	350	8	50
Pyrolysis	1600 (Pt); 1100 (Pd)	5 (Pt); 8 (Pd)	150
Atomisation	2600 (Pt); 2200 (Pd)	3	0
Cleaning	2700 (Pt); 2500 (Pd)	2 (Pt); 3 (Pd)	0

## Data Availability

Data supporting this study are included within the article and Supporting Materials.

## Supplementary Materials

The following supporting information can be downloaded at: https://www.mdpi.com/article/10.3390/molecules29245987/s1, Figure S1: Equilibrium time of the sorption of Pt and Pd by original and aged microplastics in Milli-Q water or lake water.
