# Supplementary material for: Sorption of Platinum and Palladium on Polyethylene Microplastics in Natural Water
Source: Molecules. 2024 Dec 19;29(24):5987. doi: 10.3390/molecules29245987 (PMC11678566; doi:10.3390/molecules29245987)
Supplement: Supplementary file 1 [file molecules-29-05987-s001.zip › molecules-3363607-supplementary.pdf]

Supplementary materials

Sorption of Platinum and Palladium on Polyethylene Microplastics in Natural Water

Sylwia Sajkowska [1,2] and Barbara Leśniewska [2]

[1] Doctoral School, University of Bialystok, Ciołkowskiego 1K, 15-245 Białystok, Poland;

[2] Department of Analytical and Inorganic Chemistry, Faculty of Chemistry, University of Bialystok, Ciołkowskiego 1K, 15-245 Bialystok, Poland

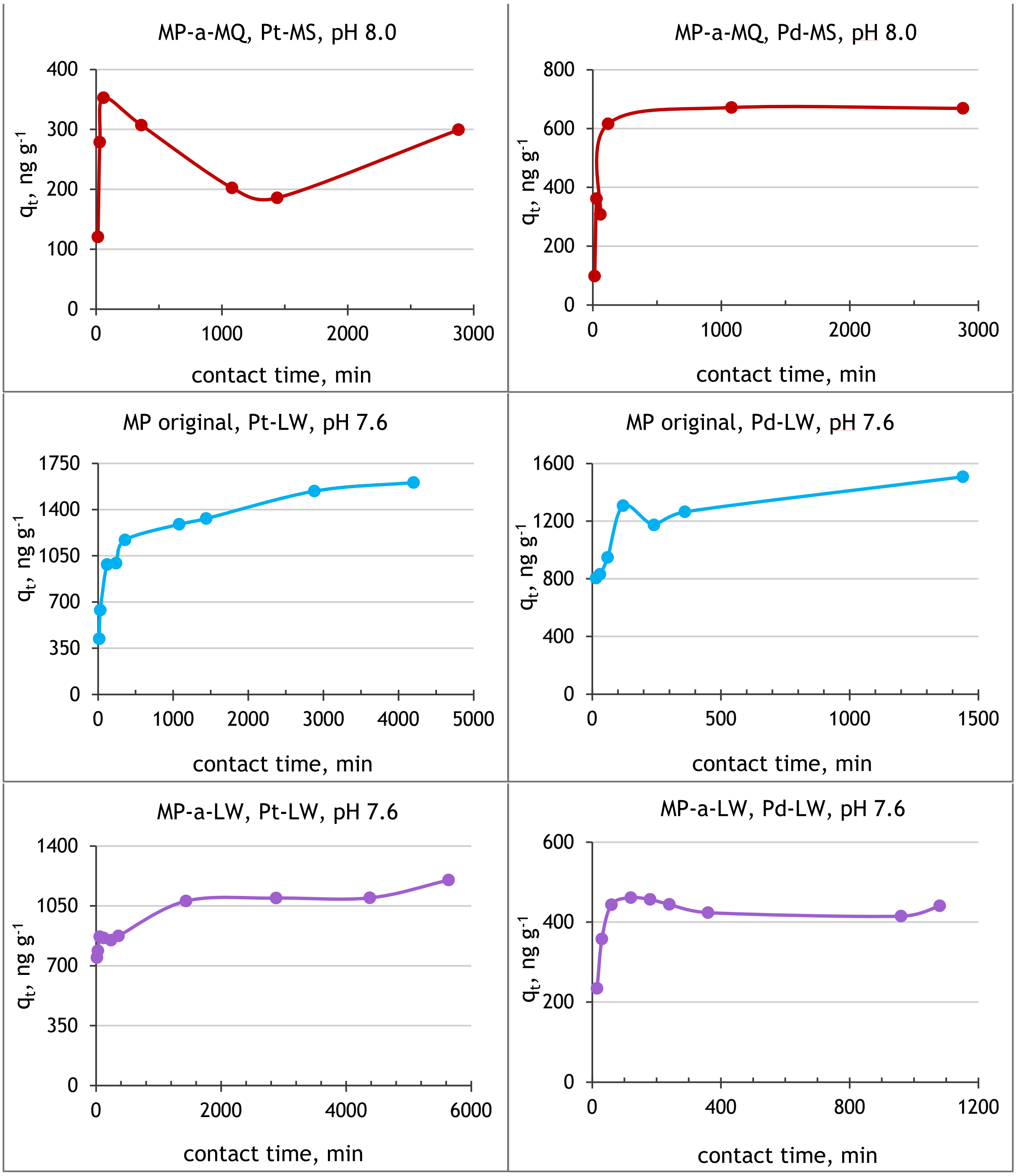

Figure S1. Equilibrium time of the sorption of Pt(IV) and Pd(II) by original and aged microplastics in Milli-Q water or lake water.
